# Impact of daily evaluation and spontaneous breathing test on the duration of pediatric mechanical ventilation: a randomized controlled trial

**DOI:** 10.1186/cc10193

**Published:** 2011-06-22

**Authors:** F Foronda, EJ Troster, JA Farias, CSV Barbas, AA Ferraro, LS Faria, A Bousso, FF Panico, AF Delgado

**Affiliations:** 1 Hospital das Clinicas da FMUSP, São Paulo - SP, Brazil

## Objective

To assess whether the combination of a daily evaluation and application of a spontaneous breathing test (SBT) could shorten the duration of mechanical ventilation (MV), as compared with weaning based on our standard of care. Secondary outcome measures included extubation failure rate and need for non-invasive ventilation (NIV).

## Methods

A prospective randomized controlled trial in two pediatric intensive care units at university hospitals in Brazil. The trial involved children between 28 days and 15 years of age who were receiving MV for at least 24 hours. Patients were randomly assigned to one of two weaning protocols. In the test group, children underwent a daily evaluation to check readiness for weaning and a SBT with pressure support of 10 cmH_2_O and PEEP of 5 cmH_2_O for 2 hours, with the SBT repeated on the next day in children failing it. In the control group, weaning was performed according to the services routine.

## Results

A total of 294 children were randomized, 155 to the test group and 139 to the control group. The time to extubation was shorter in the test group, in which the median duration of MV was 3.5 (95% CI = 3.0 to 4.0) days, in comparison with 4.7 (95% CI = 4.1 to 5.3) days in the control group (*P *= 0.0127). This significant reduction in the duration of MV in the intervention group was not associated with increased rates of extubation failure or NIV, and represents a reduction of 30% in the risk of remaining under MV (hazard ratio of 0.70).

## Conclusion

In children under MV for more than 24 hours, a daily evaluation to check readiness for weaning combined with a SBT reduced the duration of MV, without increasing the extubation failure rate or the need for NIV.

